# The effects of plyometric training on physical fitness and skill-related performance in female basketball players: a systematic review and meta-analysis

**DOI:** 10.3389/fphys.2024.1386788

**Published:** 2024-07-04

**Authors:** Shudian Cao, Zhaoran Wang, Jinwei Guo, Soh Kim Geok, He Sun, Jia Liu

**Affiliations:** ^1^ School of Physical Education, Xihua University, Chengdu, China; ^2^ School of Physical Education, Qingdao University, Qingdao, China; ^3^ Sports Department, Gaomi No. 1 Middle School, Gaomi, China; ^4^ Faculty of Educational Studies, University Putra Malaysia, Putra, Malaysia; ^5^ School of Physical Education, Henan University, Zhengzhou, China; ^6^ Department of Physical Education, Yuncheng University, Yuncheng, China

**Keywords:** plyometrics, jumps, power, agility, shooting, passing

## Abstract

**Objective:**

This study aims to analyze the effects of plyometric training (PT) on physical fitness and skill-related performance in female basketball players.

**Method:**

Five databases, including Web of Science, Scopus, PubMed, EBSCOhost, and Google Scholar, were used to select articles published up to 20 December 2023, using a combination of keywords related to PT and female basketball players. The risk of bias and the certainty of evidence in included articles were assessed using the Cochrane risk of bias (RoB2) tool and “The Grading of Recommendations Assessment, Development, and Evaluation” (GRADE).

**Results:**

Ten studies were included for the systematic review, and eight for the meta-analysis, totalling 246 female basketball players aged 14.5–22.5 years. Most of these players were highly trained. Most of the included studies exhibited concerns regarding the risk of bias. The PT programs lasted 4–8 weeks, conducted 2–3 sessions per week, with sessions lasting 20–90 min and including 29–190 jumps. In the systematic review, most studies showed that PT significantly improved performance in countermovement jump (CMJ), squat jump (SJ), Sargent jump, standing long jump, lateral hop, medicine ball throw, *t*-Test, Illinois agility, lane agility drill, linear 20-m sprint, stable and dynamic leg balance, dribbling, passing, shooting, and various basketball-specific tests, as well as increased muscle volume and thigh cross-sectional area. However, some studies showed PT to induce no significant changes in performance during CMJ, *t*-Test, Illinois agility, knee extensor/flexor strength, linear sprint, and single leg balance tests. In the meta-analysis, CMJ height (ES = 0.37; *p* = 0.036), vertical jump (VJ) peak power (ES = 0.57; *p* = 0.015), VJ peak velocity (ES = 0.26; *p* = 0.004), and *t*-Test performance time (ES = 0.32; *p* = 0.004) were significantly improved with small effects following PT.

**Conclusion:**

The effect of PT on performance in female basketball players was mixed. Most studies indicated that PT could improve various measures of physical fitness and skill-related performance, but performance remained unchanged in some tests. More studies with established tests are needed to investigate the effect of PT on female basketball players in the future.

**Systematic Review Registration:**

https://inplasy.com/, Identifier INPLASY2023120078.

## 1 Introduction

Basketball is a dynamic sport requiring numerous high-intensity actions to execute game techniques and tactics (Mancha-Triguero et al., 2019). Players with high levels of physical fitness, including agility, power, and endurance, can perform well with limited recovery time (Rodríguez-Fernández et al., 2023). Several fitness training methods have been employed for basketball players, such as resistance, core, functional, game-based conditioning, and high-intensity interval training (Cao et al., 2024). For instance, Luo et al. (2023) reported that core training could improve overall athleticism (e.g., sprinting, jumping, balance) and skill performance (e.g., shooting, dribbling, passing) in basketball players (Luo et al., 2023). Usgu et al. (2020) suggested that functional training could enhance performance-related parameters such as strength, jump height, flexibility, and agility in basketball players (Usgu et al., 2020). Among the available training methods, plyometric training (PT) is a popular choice among basketball coaching staff. For instance, all surveyed strength and conditioning coaches (n = 20) working in the National Basketball Association (NBA) indicate they use PT with their athletes (Simenz et al., 2005).

PT consists of exercises where muscles exert maximum force in short intervals to increase power (Chu, 1998). The stretch-shortening cycle (SSC) is a critical neuromuscular phenomenon underlying plyometric performance (Komi, 2003). In basketball, plyometrics aim to increase muscle power, allowing athletes to jump higher, sprint faster, and execute rapid changes in direction more effectively (Ramirez-Campillo et al., 2022)). These attributes are vital for rebounding, blocking, and shooting (Yáñez-García et al., 2022). In this regard, PT offers advantages over other methods like resistance, core, and functional training. For instance, PT targets the SSC to enhance explosive power, speed, and quickness, and also improves neuromuscular efficiency and coordination, leading to faster muscle contractions. In contrast, resistance training focuses on optimizing muscle strength and hypertrophy but may not directly improve explosive power (Lopez et al., 2021) with less focus on neuromuscular efficiency. Core training strengthens core muscles essential for stability but does not explicitly target explosive movements (Feng et al., 2024). Moreover, while functional training can improve jump performance and overall neuromuscular coordination through holistic movement patterns, it may not isolate the explosive component as effectively (Boyle, 2016; Posnakidis et al., 2022).

The effectiveness of PT has been demonstrated in many sports. For instance, Silva et al. (2019) indicated that PT could significantly improve vertical jump performance, strength, horizontal jump performance, flexibility and agility/speed in volleyball players (Silva et al., 2019). A review reported that PT improved jump height, 20-m sprint speed, and endurance in male soccer players (van de Hoef et al., 2020). Deng et al. (2022) illustrated that PT had a positive effect on maximal serve velocity and physical performance in tennis players (Deng et al., 2022). In basketball, most studies have predominantly focused on the effect of PT on male players. For example, Asadi (2013) reported that a 6-week in-season PT had positive effects for improving power and agility performance in male basketball players (Asadi, 2013). Huang et al. (2023) showed that PT could increase muscle volume in the lower limbs and legs, increase the rate of force development, and shorten the jumping time, thereby enhancing explosive strength in male basketball players (Huang et al., 2023). However, it may not be appropriate to directly apply the training effects observed in males to females due to biological differences, such as substrate metabolism and skeletal muscle fatigability (Ziv and Lidor, 2010; Landen et al., 2023). Additionally, menstrual-related factors could affect female basketball players’ performance (Gasperi et al., 2023). Finally, male players typically have higher muscle mass and greater muscle fiber cross-sectional area compared to female players (Jones et al., 2008; Bartolomei et al., 2021). Therefore, female players may experience less absolute muscle hypertrophy and strength gain from similar PT protocols.

Some systematic reviews and meta-analyses have shown that PT has a positive effect on male athletes (Ramirez-Campillo et al., 2020; van de Hoef et al., 2020; Čaprić et al., 2022), but few reviews have focused on female athletes. For instance, Pardos-Mainer et al. (2021) reported that PT significantly improved vertical jump, linear sprint, and change of direction (COD) performance more than strength training in female soccer players (Pardos-Mainer et al., 2021). Moran et al. (2019) and Stojanović et al. (2017) showed that PT effectively improves vertical jump performance in female athletes from various sports (Stojanović et al., 2017; Moran et al., 2019). However, these reviews are not specific to basketball. In this regard, Ramirez-Campillo et al. (2022) conducted a meta-analysis examining the effect of PT on physical fitness in basketball players but focused on a limited range of attributes including muscle power, linear speed, change of direction speed, balance performance, and muscle strength. In this study, there was a notable underrepresentation of studies specifically focusing on female basketball players. Most research has either mixed-sex samples or predominantly male samples, leading to a lack of targeted data on how female athletes uniquely respond to PT (Ramirez-Campillo et al., 2022). Moreover, the effect of PT on wider physical fitness attributes such as flexibility, as well as skill-related performance such as shooting, passing, and dribbling were not provided in this previous meta-analysis, creating a need to synthesise findings in this area given these are crucial elements of basketball performance. Therefore, the present systematic review and meta-analysis aimed to comprehensively investigate the effects of PT on physical fitness and skill-related performance among female basketball players.

## 2 Method

### 2.1 Protocol and registration

This study adhered to the Preferred Reporting Items for Systematic Reviews and Meta-Analyses (PRISMA 2020) guidelines (Page et al., 2021). It was registered on 19 December 2023, on the Platform of Registered Systematic Review and Meta-analysis Protocols (INPLASY2023120078).

### 2.2 Eligibility criteria

In accordance with the PICOS framework (Table 1) (Amir-Behghadami and Janati, 2020), the inclusion criteria were: 1) full-text articles published in English; 2) studies involving healthy female basketball players with no restrictions on age or skill level; 3) plyometric training (upper and/or lower limb) as the intervention in the experimental group; 4) control groups that did not undergo a PT program, or studies without control groups; 5) outcome measures that included basketball skill-related performance (e.g., shooting, passing, dribbling) or physical fitness (e.g., jump, change of direction, sprint, muscle strength); 6) randomized controlled trials (RCTs). The exclusion criteria were: 1) review articles; 2) studies recruiting male players either solely or combined with female players with data not reported separately; 3) studies that did not include a plyometric intervention or combined it with other interventions; 4) unpublished studies.

**TABLE 1:** Inclusion criteria according to the PICOS.

Items	Detailed inclusion criteria
Population	Female basketball players without injuries
Intervention	Plyometric training
Comparison	Two or more groups and single-group trials
Outcome	basketball skill-related performance (e.g., shooting, passing, dribbling) or physical fitness (e.g., jump, change of direction, sprint, muscle strength)
Study designs	RCTs

*Note*. RCTS, randomised controlled trials.

### 2.3 Information sources and search strategy

The search was conducted on 20 December 2023. The following databases were utilised: Web of Science, Scopus, PubMed, EBSCOhost, and Google Scholar (Table 2). The search terms included plyometric* OR “stretch-shortening cycle” OR “jump training” OR “jump exercise*” AND Female* OR wom?n OR girl* and basketball. Additionally, the references within the included studies were also screened.

**TABLE 2:** Number of hits for the complete search strategy for the databases.

Database	Complete search strategy	Hits 20 December 2023
Web of Science	((AB = (Plyometric* OR “stretch–shortening cycle” OR “jump training” OR “jump exercise*“)) AND AB = (Female* OR wom?n OR girl*)) AND AB = (Basketball)	43
Scopus	TITLE-ABS-KEY (plyometric* OR “stretch–shortening cycle” OR “jump training” OR “jump exercise*" AND female* OR wom?n OR girl* AND basketball)	85
PubMed	((Plyometric* [Title/Abstract] OR “stretch–shortening cycle” [Title/Abstract] OR “jump training” [Title/Abstract] OR “jump exercise*" [Title/Abstract]) AND (Female* [Title/Abstract] OR wom?n [Title/Abstract] OR girl* [Title/Abstract])) AND (Basketball [Title/Abstract])	10
EBSCOhost	AB (Plyometric* OR “stretch–shortening cycle” OR “jump training” OR “jump exercise*") AND AB (Female* OR wom?n OR girl*) AND AB Basketball	17

### 2.4 Study selection

First, duplicates were eliminated using Endnote software (X20, Thomson Reuters, New York City, NY, United States). Subsequently, two independent authors (SC and JL) screened the titles, abstracts, and full texts based on inclusion, exclusion, and PICOS criteria. Another author (HS) then double-checked the results and resolved any discrepancies through discussions with a third author (SKG) to reach the final decision. The role of each investigator was defined according to their academic titles. SPSS software (IBM Corp. Released 2022. IBM SPSS Statistics for Macintosh, Version 29.0. Armonk, NY: IBM Corp.) was used to calculate the Kappa statistic to determine the selection agreement (Narducci et al., 2011).

### 2.5 Data extraction

Following the selection of studies, specific data were extracted by the authors (SC and JL), including: 1) participant characteristics (age, height, body mass, playing level, and training experience); 2) intervention; 3) comparison (control group); 4) intervention characteristics (training content, program length, frequency, session duration, training volume, time of season); 5) assessment tests; and 6) outcomes (Table 3). Another author (HS) reviewed the information in the Microsoft Excel spreadsheet (XP professional edition; Microsoft, Redmond, WA, United States) for accuracy.

**TABLE 3:** Data extraction from included articles.

References	Participants characteristics	Intervention	Control	Characteristics of intervention				Measurements	Outcome	
				Train content	L/F/D	Volume (GC)	Season		Time	Groups
Vescovi et al. (2008)	N = 20; TB: 3 years at least EG: A = 20.3 ± 1.2 years, H = 168.4 ± 14.4 cm, BM = 66.9 ± 9.2 kg CG: A = 19.9 ± 1.6 years, H = 171.0 ± 15.2 cm, BM = 64.8 ± 9.1 kg PL: developmental	PT	No training	Wall jumps, tuck jumps, broad jumps, squat jumps, side-to-side cone jumps, front-to-back cone jumps, 180° jumps, bound in place, vertical jumps, bound for distance, scissor jumps, side-to-side mattress jumps, front-to-back mattress jumps, single-leg distance jump, jump in to bound	L: 6 weeks F: 3 sessions/week D: 45–60 min	150–190 per session 3,165 in total	NR	CMJ: height, peak power, average power, peak velocity	EG: all ↔ CG: all ↔	All ↔
Attene et al. (2015)	N = 36; TB: NR A = 14.9 ± 0.9 years, H = 164.0 ± 7.6 cm, BM = 54.0 ± 8.7 kg PL: national	PT	Basketball technical training	Front obstacle jumps with knees bending, front obstacle jumps without knees bending, countermovement and jump onto 50-cm box, drop jump from 40-cm box, lunge jump	L: 6 weeks F: 2 sessions/week D: 20 min	64–126 per session 1,120 in total	NR	CMJ: height, power, strength, speed; SJ: height, power, max power, strength, speed	EG: all ↑ CG: all ↔	CMJ and SJ ↑ in EG vs. CG
McCormick et al. (2016)	N = 14; TB: NR A = 16.0 ± 0.8 years, H = 171.9 ± 6.7 cm, BM = 60.6 ± 7.9 kg PL: national	EG1: Frontal-plane PT EG2: sagittal-plane PT	N/A	EG1: ankle jumps, squat jumps and stick, single-leg hop and stick, squat jump, single-leg hop, broad jump, split squat jump, tuck jumps EG2; side-to-side ankle jumps, lateral jump and stick, ice skater drill, lateral hop and stick, side-to-side jumps, lateral hop, lateral jump and bounce, ice skater drill, zig-zag tuck jumps	L: 6 weeks F: 2 sessions/week	96–120 per session 1,296 in total	Off-season	CMJ: height; SLJ: right leg distance, left leg distance LH: distance LST: right leg test, left leg test	EG1: all ↑ EG2: all ↑	CMJ ↓ in EG1 vs. EG2; left leg LH, left leg LST ↑ in EG1 vs. EG2; SLJ, right leg LH, right leg LST ↔ in EG1 vs. EG2
Sedaghati (2018)	N = 24; TB: 2.6 years 2.55–2.60 years EG: A = 20.3 ± 2.3 years, H = 164.0 ± 2.4 cm, BM = 63.3 ± 3.3 kg CG: A = 21.2 ± 2.8 years, H = 164.9 ± 4.2, BM = 68.3 ± 5.5 kg PL: developmental	PT	Routine training	squat jumps, ring square jumps, high-knee jumps, side and forward hopscotch, jumping along rings, pair jumping on steps, side pair jumping on steps, zig-zag jumps	L: 8 weeks F: 3 sessions/week D: 60 min	Not clear	NR	Dynamic balance test: dominant foot, non-dominant foot	EG: all ↑ CG: all ↔	Balance ↑ in EG vs. CG
Cherni et al. (2019)	N = 26; TB: 10.8 years EG: A = 20.9 ± 2.6 years, H = 172 ± 6.0 cm, BM = 65.1 ± 8.8 kg CG: A = 21.0 ± 3.0 years, H = 173 ± 7.24 cm, BM = 67.3 ± 10.6 kg PL: national	PT	Routine training	Bounding jumps, 0.4-m hurdle jumps, 0.4-m drop jumps	L: 8 weeks F: 2 sessions/week	72–126 per session 1,584 in total	In-season	*t*-Test; eyes open or closed under stable or dynamic conditions	EG: all ↑ CG: all ↔	All ↑ in EG vs. CG
Meszler and Váczi (2019)	N = 18; TB: 5 years EG: A = 15.8 ± 1.2 years, H = 176.4 ± 8.6 cm, BM = 63.5 ± 8.6 kg CG: A = 15.7 ± 1.3 years, H = 177.5 ± 7.4 cm; BM = 66.1 ± 8.9 kg PL: national	PT	Routine training	50-cm double-leg hurdle jumps, 25-cm single-leg lateral cone jumps, single-leg forward hop, 25-cm double-leg depth jumps, 35-cm double-leg lateral cone jumps; 25-cm single-leg hurdle jumps	L: 7 weeks F: 2 sessions /week D: 20 min	40–100 per session 1,027 in total	In-season	Right or left standing average on stabilometer; *t*-Test; IAT; CMJ height; knee extensors and flexors strength	EG: CMJ ↓; others ↔ CG: knee extensor strength ↑; others ↔	NR
Cherni et al. (2020)	N = 27; TB: 10.8 years EG: A = 20.9 ± 2.4 years, H = 172.0 ± 6.0 cm, BM = 65.1 ± 8.8 kg CG: A = 21.0 ± 3 years, H = 173.0 ± 7.24 cm, BM = 67.3 ± 10.6 kg PL: international	PT	Basketball training	Bounding jumps, hurdle jumps, drop jumps	L: 8 weeks F: 2 sessions/week	72–126 per session 1,584 in total	In-season	10/20/30-m sprint; *t*-Test; SJ height CMJ height; leg and thigh muscle volume; max thigh CSA	EG: CMJ, 10/20/30-m sprint ↔; leg and thigh muscle volume, max thigh CSA; SJ, *t*-Test ↑ CG: all ↔	*t*-Test; thigh CAS ↑; others ↔
Sánchez-Sixto et al. (2021)	N = 36; TB: 5 years EG: A = 22.55 ± 3.17 years, H = 166 ± 8.0 cm, BM = 64.05 ± 11.15 kg CG: A = 22.58 ± 7.28 years, H = 169 ± 6 cm, BM = 65.77 ± 8.29 kg PL: developmental	PT	No training	Drop jumps, rebound jumps	L: 6 weeks F: 2 sessions/week; D: 35 min	29–51 per session 512 in total	In-season	CMJ: height, velocity	EG: all ↑ CG: all ↔	All ↑
Pinheiro Paes et al. (2022)	N = 21; TB: NR EG: A = 14.45 ± 0.69 years, H = 1.60 ± 0.07 cm, BM = 53.72 ± 9.01 kg CG: A = 15.30 ± 1.16 years, H = 1.60 ± 0.08 cm, BM = 59.98 ± 16.74 kg PL: national	PT	Basketball Training	CMJ, side jumps, horizontal jumps, high knee jumps, split squat jumps, serial forward hops, single leg vertical jumps, single leg lateral hops	L: 6 weeks F: 2 sessions /week D: 30–60 min	50–100 per session 540 in total	Pre-season	20-m sprint IAT	EG: 20-m sprint ↑, IAT ↔ CG: 20- sprint ↑, IAT ↔	NR
Haghighi et al. (2023)	N = 24; TB: 5.1–5.3 years EG1: A = 14.6 ± 1.5 years, H = 168.3 ± 8.7 cm, BM = 61.7 ± 10.3 kg EG2: A = 15.1 ± 1.6 years, H = 167.0 ± 5.5 cm, BM = 52.5 ± 3.0 kg CG: A = 15.1 ± 1.8 years, H = 165.8 ± 9.7 cm, BM = 56.7 ± 13.6 kg PL: national	EG1: PT EG2: HIIT	Routine training	Hurdle jumps, lateral hurdle jumps, box drills with rings, depth jumps overhead ball throws, burpees, sit up and thorws	L: 6 weeks F: 2 sessions /week	105–174 per session 1,636 in total	Pre-season	20-m sprint; Sargent jump power; medicine ball throw distance; BAST; lane agility drill; basketball-specific performance; dribbling skill; passing skill; shotting skill;	EG1: all ↑ EG2: all ↑ CG: dribbling skill, passing skill ↑; others ↔	NR

*Note*. A, age; H, height; BM, body mess; PL, playing level; TB, training background; PT, plyometric training; HIIT, high-intensive interval training; NR, not reported; CG, control group; EG, experimental group; L, length; F, frequency; D, duration; GC, ground contacts; CMJ, countermovement jump; SJ, squat jump; SLJ, standing long jump; LST, lateral shuffle test; LH, lateral hop; CSA, cross-sectional areas; BAST, basketball-based anaerobic specific test; IAT, illinois agility test; ↑, significantly positive effect (*p* ≤ 0.05); ↓, significantly negative effect (*p* ≤ 0.05); ↔, no effect (*p* > 0.05).

### 2.6 Risk of bias assessment and certainty of evidence

The Cochrane risk of bias tool (RoB 2) was employed by two authors (SC and JL) to assess the risk of bias in all included randomized controlled trials (RCTs), following the guidelines by Sterne et al. (2019) (Sterne et al., 2019). RoB two evaluates bias in five domains: bias arising from the randomization process, deviations from the intended interventions, missing outcome data, measurement of the outcome, and selection of the reported result. In case of disagreements in the risk of bias assessments, the third author (XW) resolved them. Ultimately, an overall risk of bias score was determined. The certainty of the evidence was evaluated using “The Grading of Recommendations Assessment, Development, and Evaluation (GRADE)” approach (Goldet and Howick, 2013). This assessment considers factors such as study design, risk of bias, inconsistency, indirectness, imprecision, and publication bias to determine the certainty of evidence. A summary of the findings table was generated with the assistance of GRADEpro GDT and carried out independently by two authors.

### 2.7 Statistical Analysis

In accordance with previous research, studies that provided three or more sets of baseline and follow-up data for the same variables underwent meta-analysis using Meta-analysis software (version 3.0), with a statistical significance threshold of *p* < 0.05. The meta-analysis employed the inverse-variance random-effects model to account for heterogeneity among studies. The I^2^ statistic was used to assess heterogeneity, categorized into low (<25%), moderate (25%–75%), and high (>75%) values. Effect sizes (ES) between groups were computed using Hedge’s g, and 95% confidence intervals (CIs) were reported for the ES values. Effect sizes were categorized as trivial (<0.2), small (0.2–0.6), moderate (>0.6–1.2), large (>1.2–2.0), very large (>2.0–4.0), and extremely large (>4.0) (Hopkins et al., 2009). The extended Egger’s test was used to evaluate the risk of publication bias across studies (Egger et al., 1997). A sensitivity analysis was performed when Egger’s test indicated a low *p*-value (*p* < 0.05), suggesting significant asymmetry in the funnel plot, indicating that smaller studies with non-significant or negative results might be underrepresented in the meta-analysis. A higher *p*-value (*p* ≥ 0.05) suggested that the funnel plot was symmetrical, indicating no strong evidence of missing studies based on their size and effect (Egger et al., 1997).

## 3 Results

### 3.1 Study selection

**FIGURE 1:**
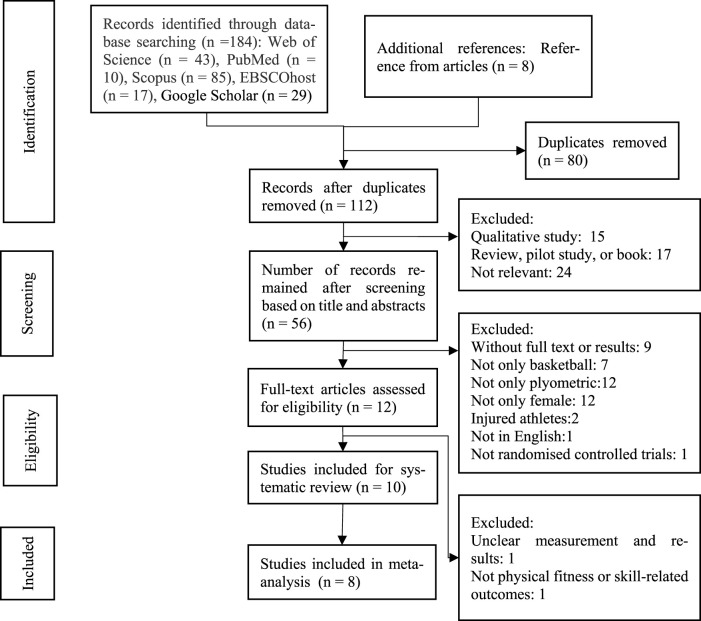
Systematic review search and screening procedure.

A total of 192 studies were initially identified through the search process, and 80 duplicates were removed using Endnote software. Following screening titles, abstracts, and full-text articles, ten articles met the criteria for inclusion in the systematic review, and eight were eligible for inclusion in the meta-analysis (Figure 1). Two articles were not included for the meta-analysis due to heterogeneity in outcomes. The Kappa statistic for agreement between authors, calculated using SPSS software, was 1.00.

### 3.2 Risk of bias assessment and certainty of evidence

**FIGURE 2:**
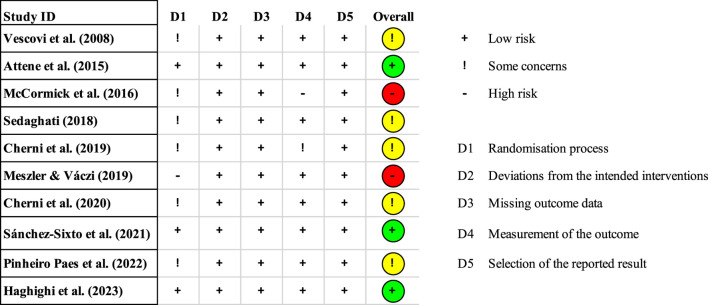
Risk of bias for each study.

**FIGURE 3:**
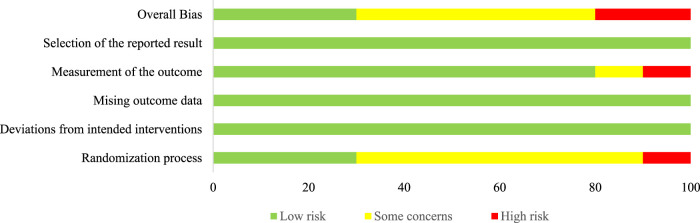
Risk of overall bias.

Figure 2 shows the risk of bias for each study according to RoB 2, and the overall risk of bias across all studies is presented in Figure 3. Notably, all included articles demonstrated a low risk of bias in the domains related to deviations from the intended interventions, missing outcome data, and selection of the reported results. However, only three studies employed proper randomization techniques. Attene et al. (2015) implemented block randomization to ensure equal group sizes. Sánchez-Sixto et al. (2021) used balanced randomization to assign participants to groups. Haghighi et al. (2023) electronically generated the randomization sequence and concealed the process until interventions were assigned. One study had limitations in its outcome measurement methodology (McCormick et al., 2016). In summary, most of the included studies exhibited concerns regarding the risk of bias. Furthermore, the summary of findings table generated using GRADEpro GDT indicated that the certainty of evidence ranged from high to very low (see Supplementary Appendix A).

### 3.3 Participant characteristics

(1) Sample Size. The ten articles comprised 246 subjects, with individual studies ranging from 14 (McCormick et al., 2016) to 36 participants (Attene et al., 2015; Sánchez-Sixto et al., 2021). The mean sample size across all studies was 24.6 participants (SD = 7.1).
(2) Age. The ages of participants varied across the studies, with the youngest participants being around 14.5 years old (Pinheiro Paes et al., 2022) and the oldest 22.6 years old (Sánchez-Sixto et al., 2021). The mean age across all studies was 18.5 years old (SD = 3.07).
(3) Playing Level. The level of players in the included studies was determined by the participant classification framework (McKay et al., 2021). Six of the studies focused on national level players (Attene et al., 2015; McCormick et al., 2016; Cherni et al., 2019; Meszler and Váczi, 2019; Pinheiro Paes et al., 2022; Haghighi et al., 2023), three examined developmental players (Vescovi et al., 2008; Sedaghati, 2018; Sánchez-Sixto et al., 2021), and only one focused on international level players (Cherni et al., 2020).

### 3.4 Intervention characteristics

The intervention characteristics of the ten articles were summarized as follows.

1. Training Program Length: The duration of PT in these studies ranged from 6 to 8 weeks.
2. Training Duration: Six studies reported the training duration of PT sessions (Vescovi et al., 2008; Attene et al., 2015; Sedaghati, 2018; Meszler and Váczi, 2019; Sánchez-Sixto et al., 2021; Pinheiro Paes et al., 2022), which varied from 20 to 60 min.
3. Training Frequency: The PT frequency across all studies ranged from two to three sessions per week.
4. Training Volume: The PT volume across nine studies ranged from 29 jumps per session (Sánchez-Sixto et al., 2021) to 190 jumps per session (Vescovi et al., 2008) and from 512 total jumps (Sánchez-Sixto et al., 2021) to 3,165 total jumps (Vescovi et al., 2008). One study did not clearly provide the training volume (Sedaghati, 2018).
5. Training Time of Season: Two studies implemented PT during the pre-season (Pinheiro Paes et al., 2022; Haghighi et al., 2023), four studies during the in-season (Cherni et al., 2019; Meszler and Váczi, 2019; Cherni et al., 2020; Sánchez-Sixto et al., 2021), one study during the off-season (McCormick et al., 2016), and three studies did not report the training time (Vescovi et al., 2008; Attene et al., 2015; Sedaghati, 2018).

### 3.5 Outcomes of systematic review

All ten included articles examined various physical fitness outcomes, including power, agility, speed, balance, and muscular strength. Only one included assessments of skill-related basketball performance (Haghighi et al., 2023).

1. Effect of PT on power-related attributes: Seven studies evaluated the impact of PT on power-related attributes, utilizing measurements such as CMJ (height, power, velocity, strength, speed) (Vescovi et al., 2008; Attene et al., 2015; McCormick et al., 2016; Meszler and Váczi, 2019; Cherni et al., 2020; Sánchez-Sixto et al., 2021), squat jump (SJ) (height, power, strength, speed) (Attene et al., 2015; Cherni et al., 2020), Sargent jump power (Haghighi et al., 2023), standing long jump distance (McCormick et al., 2016), and lateral hop distance (McCormick et al., 2016). Four studies reported significant improvements in power-related attributes following PT, indicated by increased performance in CMJ (height, power, velocity) (Attene et al., 2015; McCormick et al., 2016; Cherni et al., 2020; Sánchez-Sixto et al., 2021), Sargent jump power (Haghighi et al., 2023), standing long jump distance (McCormick et al., 2016), and lateral hop distance (McCormick et al., 2016). However, two studies found no significant difference in CMJ performance before and after PT (Vescovi et al., 2008; Cherni et al., 2020), and one study showed decreased CMJ performance following PT (Meszler and Váczi, 2019). Additionally, one study evaluated the effect of PT on medicine ball throw distance (Haghighi et al., 2023), showing significant improvement.
2. Effect of PT on linear and change of direction speed: Three studies examined the effect of PT on linear speed, including assessments of 10-m (Cherni et al., 2020), 20-m (Cherni et al., 2020; Pinheiro Paes et al., 2022; Haghighi et al., 2023), and 30-m sprint (Cherni et al., 2020) tests. Two studies reported improvements in 20-m sprint time (Pinheiro Paes et al., 2022; Haghighi et al., 2023), but one study found no significant impact on 10-m, 20-m, and 30-m sprint time (Cherni et al., 2020). Six studies used assessments such as the lateral shuffle test (McCormick et al., 2016), *t*-Test (Cherni et al., 2019; Meszler and Váczi, 2019; Cherni et al., 2020), Illinois agility test (Meszler and Váczi, 2019; Pinheiro Paes et al., 2022), and lane agility drill test (Haghighi et al., 2023) to evaluate the effect of PT on change of direction (COD) speed. Four studies reported a positive impact of PT on COD speed (McCormick et al., 2016; Cherni et al., 2019; Cherni et al., 2020; Haghighi et al., 2023), while two studies (Meszler and Váczi, 2019; Pinheiro Paes et al., 2022) found no improvement in the *t*-Test and Illinois agility test.
3. Effect of PT on muscle strength: Two articles explored the effect of PT on muscle strength. One study used assessments of knee extensors and flexors strength (Meszler and Váczi, 2019). Another study (Cherni et al., 2020) assessed the muscle volume and the cross sectional area (CSA) of the thigh, which are highly related with muscle strength (Jones et al., 2008; Akagi et al., 2009). Cherni et al. (2020) indicated that PT increased leg and thigh muscle volume and maximum thigh CSA, while Meszler and Váczi (2019) found no significant impact on knee extensors and flexors strength.
4. Effect of PT on balance: Three studies investigated the effect of PT on balance, including assessments of the dynamic balance test (Sedaghati, 2018) eyes open or closed under stable or dynamic conditions (Cherni et al., 2019), and single leg standing average on a stabilometer (Meszler and Váczi, 2019). Two studies showed that PT improved both stable and dynamic leg balance tests (Sedaghati, 2018; Cherni et al., 2019), while one study found no positive effects on single leg standing average on a stabilometer test (Meszler and Váczi, 2019).
5. Effect of PT on basketball-related skills: Only one study examined the impact of PT on basketball skill-related performance (Haghighi et al., 2023). The study reported improvements in dribbling, passing, shooting, and various basketball-specific performances in a circuit following the PT intervention.

### 3.6 Outcome of meta-analysis

Eight of the articles were analysed using meta-analysis software (version 3.0), including the effect of PT on power, agility, and speed.

#### 3.6.1 Effect of PT on CMJ height, VJ peak power, and VJ velocity

**FIGURE 4:**
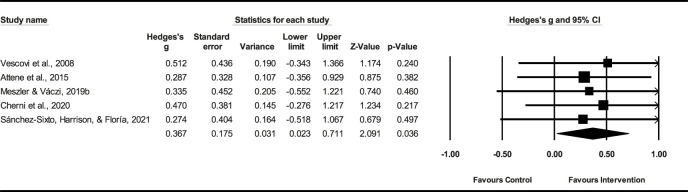
Forest plot of PT on CMJ height.

Five studies (*n* = 124) demonstrated that the PT had a small effect on CMJ height (ES = 0.37; %95 CI = 0.02–0.71; *p* = 0.036). The heterogeneity among the studies was low (*I* ^2^ = 0.0%). The Egger’s test demonstrated a *p* = 0.51 (Figure 4), indicating no significant publication bias among the studies.

**FIGURE 5:**
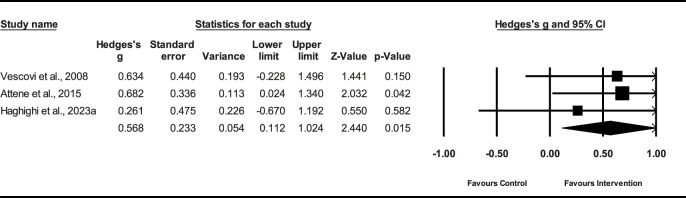
Forest plot of PT on VJ peak power.

Three studies (*n* = 72) demonstrated that the PT had a small effect on VJ peak power (ES = 0.57; %95 CI = 0.02–0.71; *p* = 0.015). The heterogeneity among the studies was low (*I* ^2^ = 0.0%). The Egger’s test demonstrated a *p* = 0.43 (Figure 5), indicating no significant publication bias among the studies.

**FIGURE 6:**
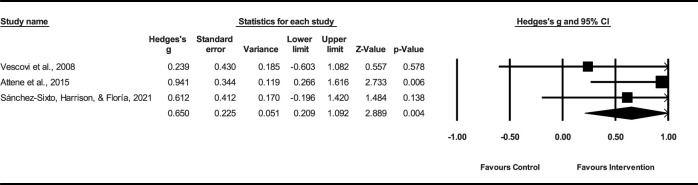
Forest plot of PT on VJ velocity.

Three studies (*n* = 79) demonstrated that the PT had a small effect on VJ peak velocity (ES = 0.26; %95 CI = 0.21–1.10; *p* = 0.004). The heterogeneity among the studies was low (*I* ^2^ = 0.0%). The Egger’s test demonstrated a *p* = 0.21 (Figure 6), indicating no significant publication bias among the studies.

#### 3.6.2 Effect of PT on *t*-Test

**FIGURE 7:**
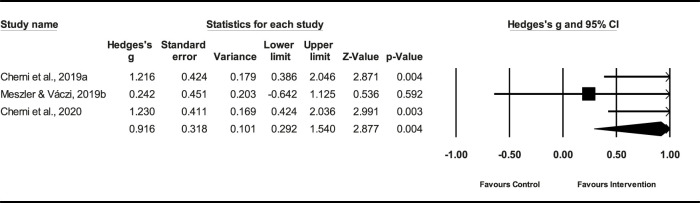
Forest plot of PT on *t*-Test.

Three studies (*n* = 70) demonstrated that the PT had a small effect on the *t*-Test (ES = 0.32; %95 CI = 0.29–1.54; *p* = 0.004). The heterogeneity among the studies was moderate (*I* ^2^ = 39.75%). The Egger’s test demonstrated a *p* = 0.20 (Figure 7), indicating no significant publication bias among the studies.

#### 3.6.3 Effect of PT on 20-m sprint

**FIGURE 8:**
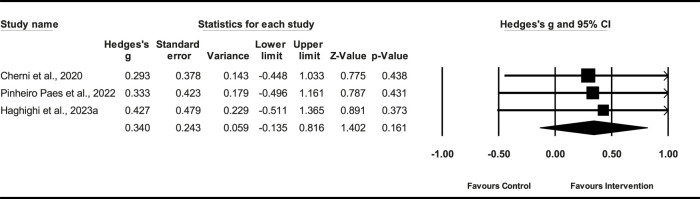
Forest plot of PT on 20-m sprint.

Three studies (*n* = 64) demonstrated that the PT had a small effect on the 20-m sprint (ES = 0.24; %95 CI = −0.135–0.816; *p* = 0.161). The heterogeneity among the studies was low (*I* ^2^ = 0.0%). The Egger’s test demonstrated a *p* = 0.12 (Figure 8), indicating no significant publication bias among the studies.

## 4 Discussion

This systematic review and meta-analysis aimed to examine the impact of PT on the physical fitness and skill-related performance of female basketball players. The systematic review revealed that most included studies reported a significantly positive effect of PT on physical fitness components, such as power, agility, speed, balance, and muscular strength. However, a few studies did not observe significant changes in certain tests, including the CMJ performance, *t*-Test, Illinois agility test, knee extensors and flexors strength test, 10, 20, and 30-m linear sprints, and single leg standing average on the stabilometer test. Notably, only one study investigated the effect of PT on basketball-specific performance, reporting significant improvements (Haghighi et al., 2023). In the meta-analysis, significant differences with small effect sizes were found in the effects of PT on CMJ height, vertical jump peak power and velocity, and the *t*-Test (*p* < 0.05), but not on the 20-m sprint (*p* > 0.05). The effects of PT on female basketball players are specifically discussed in the following sections.

### 4.1 Effect of PT on power-related attributes

Power-related attributes are crucial for basketball players due to the sport’s dynamic and multidirectional nature, which requires rapid and explosive movements (Delextrat and Cohen, 2008; Abdelkrim et al., 2010; Scanlan et al., 2011; Gonzalez et al., 2013). Four studies in the review demonstrated that PT had a significant positive effect on various types of jump performance (Attene et al., 2015; Cherni et al., 2020; Sánchez-Sixto et al., 2021) and medicine ball throw distance (Haghighi et al., 2023). These findings align with results from previous reviews (Markovic, 2007; Stojanović et al., 2017; Silva et al., 2019). Improvements in power-related performance, particularly jump ability, are highly relevant for basketball players as they are essential for executing advanced skills like rebounding, sprinting, and jump shots during games (Altavilla et al., 2018). The mechanism of PT concerning the stretch-shortening cycle (SSC) can explain the improvement in power. Plyometric exercises engage the SSC, allowing muscles to store elastic energy during the eccentric phase (muscle lengthening) and release it quickly during the concentric phase (muscle shortening) (Chu, 1998; Chu and Meyer, 2013). This results in more powerful and explosive movements. Additionally, the high-intensity, rapid nature of plyometric exercises enhances the nervous system’s ability to recruit muscle fibers more effectively, increasing the speed and coordination of muscle contractions (Zubac et al., 2019; Galay et al., 2021).

However, two studies indicated that PT did not enhance CMJ performance (Vescovi et al., 2008; Cherni et al., 2020). These divergent results may be attributed to specific participant characteristics, such as their prior experience with PT and the training content (Moran et al., 2017; Stojanović et al., 2017). Compared to the studies that showed the significant improvement of PT on power-related attributes, participants in Vescovi et al. (2008) had over 3 years of training experience, which was the least except for two studies that did not report the experience (Vescovi et al., 2008). Three years of training experience may not be sufficient to develop the foundational strength and technical skills necessary for effective plyometric exercises (Sole et al., 2022). Players still developing neuromuscular coordination might struggle to achieve optimal muscle activation patterns required for maximal power gains (Bompa and Carrera, 2015). Cherni et al. (2020) showed that PT significantly improved SJ but not CMJ performance, which runs counter to the previous study that reported somewhat greater positive effects in CMJ than SJ performance (Stojanović et al., 2017). More studies are needed to explore the reasons for the divergence.

Additionally, one study reported adverse effects of PT on CMJ height (Meszler and Váczi, 2019). This might be due to the PT program being implemented during the in-season basketball competition. During the season, players already experience physical and mental fatigue from regular practices, games, and travel. Adding PT might overload their recovery capacity, leading to cumulative fatigue and decreased performance (Chelly et al., 2010). The busy game schedule during the season may not provide sufficient recovery time between PT sessions (Asadi, 2013), leading to inadequate muscle recovery and reduced benefits from PT. PT should be periodized with specific attention to high-load and low-load phases to maximize performance gains while minimizing fatigue (Chelly et al., 2010).

### 4.2 Effect of PT on COD speed

COD speed is critical in basketball. For instance, defenders rely on COD ability to stay in front of their opponents, adjust to sudden movements, and close out on shooters (Ivanović et al., 2022). Good COD speed ability, combined with proper technique, can help reduce the risk of injuries (Dos’ Santos et al., 2021). The results of PT on COD speed were inconsistent, aligning with results from previous reviews (Asadi et al., 2016; Sole et al., 2021). Most studies demonstrated that PT had a significantly positive effect on the lateral shuffle test (McCormick et al., 2016), *t*-Test (Cherni et al., 2019; Cherni et al., 2020), and lane agility drill (Haghighi et al., 2023) among female basketball players. The lateral shuffle test assesses an athlete’s lateral movement agility (Patowary and Das, 2023). The *t*-Test assesses forward, lateral, and backward movement, providing a more comprehensive evaluation of agility compared to the lateral shuffle test (Keš et al., 2020). The lane agility drill also includes a combination of forward, lateral, and backward movements, similar to the *t*-Test but within a confined space (Čaušević et al., 2023). Neural adaptations, including increased recruitment of motor units (Miller et al., 2006; Markovic and Mikulic, 2010; Asadi et al., 2016) and enhanced neural drive to agonist muscles induced by PT, can improve cutting skills, allowing players to exhibit better body control and skill performance during games (Markovic and Mikulic, 2010).

However, two studies did not indicate a significant difference in the *t*-Test (Meszler and Váczi, 2019) and the Illinois agility test (Pinheiro Paes et al., 2022). The meta-analysis also revealed a small PT effect size (ES = 0.32) on the *t*-Test. Aside from the issue of PT being conducted in-season (Meszler and Váczi, 2019), the lower number of jumps per session (50–100 jumps) in Pinheiro Paes et al. (2022) compared to others (72–174 jumps) might be another reason. Volume plays a critical role in neuromuscular adaptation, and if the jump count is too low, it may not generate sufficient muscle engagement or neural activation (Ramírez-Campillo et al., 2013). Additionally, both the *t*-Test and Illinois agility test combine lateral movements, forward sprints, and backward runs, which demand high levels of coordination in multiple planes of motion (Raya et al., 2013). However, most plyometric exercises in the included studies primarily focused on the sagittal plane, such as vertical jumps, box jumps, and bounding. These exercises are excellent for improving power and explosiveness in forward and backward movements but may not fully address the lateral and rotational movements required in the *t*-Test and Illinois agility test (Weltin et al., 2017).

### 4.3 Effect of PT on linear speed

In basketball competitions, players sprinting down the court to score quickly on offense or stop a fast break on defense need excellent linear speed (Scanlan et al., 2014). In two studies, results showed that PT significantly improved 20-m sprint performance (Pinheiro Paes et al., 2022; Haghighi et al., 2023). The meta-analysis indicated a small effect size (ES = 0.24) of PT on the 20-m sprint. These results are in line with those reported in a previous review showing PT was effective in improving sprint performance (ES = 0.37) (de Villarreal et al., 2012). The improvement in linear speed performance can be explained in several ways. First, specific PT exercises such as depth jumps, box jumps, and bounding enhance the explosive power of the lower body (Aksović et al., 2021), aiding in rapid force generation at the start of the sprint (de Villarreal et al., 2012). Additionally, plyometrics improves the SSC, which involves a rapid muscle stretch followed by a quick contraction (Galay et al., 2021). Enhanced SSC efficiency maximizes force production with minimal ground contact time, crucial for fast acceleration (de Villarreal et al., 2012). Moreover, participants in the studies by Haghighi et al. (2023) and Pinheiro Paes et al. (2022) implemented PT during the pre-season. During this period, there is less pressure from games or competitions, allowing athletes to focus on training quality without the risk of fatigue affecting in-season performance.

However, one study found no impact of PT on 10-m, 20-m, and 30-m sprint speed (Cherni et al., 2020). This could be due to the PT being implemented during the in-season, the training content including only three types of jumps, and the elite players potentially reaching a performance plateau. These reasons have already been discussed previously.

### 4.4 Effect of PT on muscle strength

Strong muscles, particularly in the legs and core, provide the power necessary for high jumps, quick acceleration, and effective pivoting in basketball competition (Cabarkapa et al., 2022). However, only two included studies investigated the effects of PT on muscle strength (Cherni et al., 2019; Meszler and Váczi, 2019), and their findings were contradictory. Cherni et al. (2020) demonstrated that PT increased leg and thigh muscle volume and maximum thigh CSA, leading to improvements in strength. This aligns with a previous review that reported PT has a significant positive effect on maximal strength compared to other training methods such as weight training, eccentric training, and isometric training (De Villarreal et al., 2010). Plyometric exercises improve the communication between the nervous system and muscles, allowing for more coordinated and rapid muscle contractions (Chimera et al., 2004). This efficiency means that muscles can apply more force in a controlled and effective manner. Additionally, plyometrics enhances tendon stiffness, allowing tendons to store and release more elastic energy during explosive movements (Fouré et al., 2010; Ramírez-delaCruz et al., 2022). This contributes to greater force production and muscle strength.

In contrast, Meszler and Váczi (2019) indicated that PT did not impact knee extensors and flexors strength. This discrepancy may be due to the participants in this study being younger than 16 years old. At this age, the musculoskeletal system is still maturing, and they have not fully developed the hormonal environment that supports muscle growth to the same degree as adults (Fink et al., 2018). Lower levels of testosterone and growth hormone reduce the potential for significant muscle growth (Gharahdaghi et al., 2021).

### 4.5 Effect of PT on balance

Good balance provides a solid, upright, and steady foundation for playing basketball. This stability supports various aspects of basketball, including running, defending, shooting, dribbling, passing, and rebounding (Halabchi et al., 2020). Additionally, good balance can help reduce the risk of sustaining injuries (Sañudo et al., 2019; Crossley et al., 2020). Two included articles have shown that PT improved both stable and dynamic leg balance (Sedaghati, 2018; Cherni et al., 2019), which is in lines with results from previous review (Ramachandran et al., 2021). Neuromuscular adaptations and proprioception enhancement are key factors contributing to improved balance. Specifically, plyometric exercises involve rapid stretching and contracting of muscles, enhancing the neuromuscular system’s ability to respond quickly and efficiently (Huang et al., 2021). This improved neuromuscular control is crucial for maintaining balance during dynamic movements (Piirainen et al., 2014). Furthermore, plyometrics often require athletes to perform exercises on unstable surfaces or in challenging positions, improving proprioception, which is the body’s ability to sense its position and movement in space (Alikhani et al., 2019; Zhou et al., 2022). Better proprioception leads to better balance and stability.

### 4.6 Effect of PT on basketball-related skills

Haghighi et al. (2023) demonstrated that PT can lead to improvements in dribbling, passing, shooting skills, and other basketball-specific performance measures. Basketball shooting skills rely on physical fitness such as upper body and leg strength for generating shot power, and core stability for balance and control (Candra et al., 2018; Aksović et al., 2020; Cabarkapa et al., 2022; Jing, 2023). Upper body strength, especially in the shoulders, arms, and chest, is essential for generating the necessary power to shoot the basketball, which is particularly important for long-range shots such as three-pointers (Cabarkapa et al., 2022). Explosive power in the legs and core allows for a quick and high jump, which is essential for creating space from defenders and getting the shot off cleanly (Candra et al., 2018; Aksović et al., 2020). Good balance provides a solid foundation for shooting. It allows players to set their feet properly, align their body towards the basket, and execute the shot with proper form (Jing, 2023). The improvement of these physical fitness attributes in included studies could explain the effectiveness of PT on shooting skills. Strength, COD ability, and balance are important in executing basketball passing skills (Nikolaos et al., 2012; Spiteri et al., 2015). The upper body strength allows players to deliver passes over varying distances with the necessary force (Ahmed, 2013). Good COD ability enables players to position themselves correctly for making effective passes (Spiteri et al., 2015). Being able to move swiftly and change directions helps in avoiding defenders and creating passing lanes (Spiteri et al., 2015). Balance is essential when making passes while in motion or after a quick COD (Nikolaos et al., 2012; Fisek and Agopyan, 2021). Basketball dribbling skills are not only related to ball control, vision, and court awareness but also depend on various physical fitness factors such as hand speed, coordination, changes in pace and speed, and directional control (Ferioli et al., 2020; Vencúrik et al., 2021). The present study already shows that PT improved physical attributes, including explosiveness and speed, which are essential for quick changes of direction and acceleration while dribbling.

However, without sufficient research focusing on these areas, it is challenging to draw comprehensive conclusions about the benefits of PT on skill-related performance in female players. Male and female athletes may respond differently to the same training protocols due to physiological and hormonal differences (Ziv and Lidor, 2010; Gasperi et al., 2023; Landen et al., 2023). Understanding these unique responses is crucial for developing optimized and effective training programs tailored to female athletes. Therefore, comprehensive research involving multiple studies is necessary to understand the full impact of PT on basketball skills in female players. This line of research should include examining different types of plyometric exercises, training durations, and their specific effects on various basketball skills.

## 5 Limitations

Several limitations should be considered in this study. Firstly, the limited number of studies resulted in a relatively small amount of data available for the meta-analysis. This study did not separately analyze the results according to age and playing level categorization, such as adolescents and adults, international level and national level, due to the limited number of articles, which may affect the analysis. Finally, while the included studies provided comprehensive details about the PT program, a few of them did not specify the training content of the control group. This lack of information could introduce bias in the results, and also limit the practical application of the program for players.

## 6 Conclusion

This review with meta-analysis provides evidence on the effects of PT on female basketball players. Most of the included articles indicated that PT significantly improved jumping and throwing skills, sprinting and cutting skills, muscle properties, balance, and skill-related performance among female basketball players. However, a few studies showed no significant difference on some tests, including the CMJ test, *t*-Test, Illinois agility test, knee extensors and flexors strength test, 10, 20, and 30-m sprint tests, and single-leg standing average on stabilometer test.

## 7 Practical implications

While the current evidence supports the efficacy of PT in enhancing physical fitness and some skill-related performance measures in female basketball players, the limited number of studies highlights the need for further research. More comprehensive and focused studies are required to fully understand the impact of PT on skill-related performance, ensuring that training programs can be optimized for female athletes. Given the preliminary evidence supporting the potential benefits of PT, predominantly encompassing various types of jump drills, provided in this review, basketball coaches and trainers working with female players should consider including this form of training within their annual plan. In doing so, basketball coaches and trainers should properly manage the training load in an appropriately periodized manner to ensure physical fitness and skill-related performance are continuously optimized across the season. As more evidence is provided on this topic in female basketball players, the most beneficial PT drills for certain physical fitness attributes and skills may be elucidated to provide further specificity in training prescription.

## Data Availability

The original contributions presented in the study are included in the article/Supplementary Material, further inquiries can be directed to the corresponding author.
