# Supplementary material for: The effects of plyometric training on physical fitness and skill-related performance in female basketball players: a systematic review and meta-analysis
Source: Front Physiol. 2024 Jul 4;15:1386788. doi: 10.3389/fphys.2024.1386788 (PMC11254773; doi:10.3389/fphys.2024.1386788)
Supplement: Supplementary file 1 [file Table1.docx]

**Appendix A**

| **Plyometric training compared to no plyometric training for physical fitness and basketball performance on female basketball players** | | | | | | |
| --- | --- | --- | --- | --- | --- | --- |
| **Certainty assessment** | | | | | | |
| **Participants (studies)** | **Risk of bias** | **Inconsistency** | **Indirectness** | **Imprecision** | **Publication bias** | **Overall certainty of evidence** |
|  |  |  |  |  |  |  |
| **Power** | | | | | | |
| 175 (7 RCTs)  Vescovi et al., 2008  Attene et al., 2015  McCormick et al., 2016  Meszler & Váczi, 2019  Cherni et al., 2020  Sánchez-Sixto et al., 2021  Haghighi et al., 2023 | serious^a^ | serious^b^ | not serious | serious^c^ | none | ⨁◯◯◯ Very low |
| **Agility** | | | | | | |
| 130 (6 RCTs)  McCormick et al., 2016  Cherni et al., 2019  Meszler & Váczi, 2019  Cherni et al., 2020  Pinheiro Paes et al., 2022  Haghighi et al., 2023 | serious^a^ | serious^b^ | not serious | serious^c^ | none | ⨁◯◯◯ Very low |
| **Muscle strength** | | | | | | |
| 44 (2 RCTs)  Cherni et al., 2019  Meszler & Váczi, 2019 | serious^a^ | serious^b^ | not serious | serious^c^ | none | ⨁◯◯◯ Very low |
| **Speed** | | | | | | |
| 72 (3 RCTs)  Cherni et al., 2020  Pinheiro Paes et al., 2022  Haghighi et al., 2023 | serious^a^ | serious^b^ | not serious | serious^c^ | none | ⨁◯◯◯ Very low |
| **Balance** | | | | | | |
| 68 (3 RCTs)  Sedaghati, 2018  Cherni et al., 2019  Meszler & Váczi, 2019 | serious^a^ | serious^b^ | not serious | serious^c^ | none | ⨁◯◯◯ Very low |
| **Basketball skill performance** | | | | | | |
| 24 (1 RCT)  Haghighi et al., 2023 | not serious | not serious | not serious | not serious | none | ⨁⨁⨁⨁ High |

**CI:** confidence interval; **MD:** mean difference

#### Explanations

a. Some included articles had some concerns or high risk of bias.

b. There were different outcomes among studies.

c. The sample size was not calculated by the appropriate method.
